# Wave-driven electron inward transport in a magnetic nozzle

**DOI:** 10.1038/s41598-022-24202-9

**Published:** 2022-12-05

**Authors:** Kazunori Takahashi, Christine Charles, Rod W. Boswell

**Affiliations:** 1 Department of Electrical Engineering, Tohoku University, Sendai, 980-8579 Japan; 2 Space Plasma, Power and Propulsion Laboratory, Research School of Physics, The Australian National University, Canberra, ACT 2601 Australia; 3 Interdisciplinary Research Center for Non-equilibrium Plasma, Tohoku University, Sendai, 980-8579 Japan

**Keywords:** Plasma physics, Space physics

## Abstract

Plasma flows in divergent magnetic fields resembling a magnetic nozzle can be found over wide scales ranging from astrophysical objects to terrestrial plasma devices. Plasma detachment from a magnetic nozzle is a frequent occurrence in natural plasmas, e.g., plasma ejection from the Sun and release from the Sun’s magnetic field, forming the solar wind. Plasma detachment has also been a challenging problem relating to space propulsion devices utilizing a magnetic nozzle, especially the detachment of the magnetized electrons having a gyro-radius smaller than the system’s scale is required to maintain zero net current exhausted from the system. Here we experimentally demonstrate that a cross-field transport of the electrons toward the main nozzle axis, which contributes to neutralizing the ions detached from the nozzle, is induced by the spontaneously excited magnetosonic wave having the frequency considerably higher than the ion cyclotron frequency and close to the lower hybrid frequency, driving an **E** × **B** drift that only effects the electrons. Wave-induced transport and loss have been one of many important issues in plasma physics over the past several decades. Conversely, the presently observed electron inward transport has a beneficial effect on the detachment by reducing the divergence of the expanding plasma beam; this finding will open a new perspective for the role of waves and instabilities in plasmas.

## Introduction

Plasma flows in diverging magnetic fields, sometimes called a magnetic nozzle (MN), have been discovered to exist over wide scales ranging from astrophysical phenomena^1,2^, solar plasmas^3^, the Earth’s magnetosphere and ionosphere^4^, and terrestrial plasma devices^5^. During expansion, the plasmas can gain or lose energy and momentum via various static and dynamic phenomena, e.g., waves, turbulence, and electromagnetic forces. Processes in the MN play a significant role in the development of new-type space propulsion devices consisting of the high density plasma source and the MN, where ions are accelerated mainly into the axial direction by spontaneous steady-state electric fields and a Lorentz force due to the internal electron current and the applied magnetic field directs their momentum towards the axial direction^6–13^. Since most of the input electric power in the laboratory experiments is typically coupled to the electrons, their cooling and transport in the MN are essential to an in-depth understanding of energy transport and conversion processes^14–18^.

Eventually, the plasma has to become detached from the magnetic field lines to generate the thrust by the electric propulsion device; otherwise, the charged particles would return to the thruster along the closed magnetic field lines. A few scenarios of the detachment have been proposed, e.g., magnetohydrodynamic and unmagnetization detachment^19–23^. The ions are often unmagnetized in the MN, with trajectories dominated by the steady-state electric fields and deviating considerably from the magnetic field lines^9^, while the electron Larmor radius is much smaller than the plasma scale; hence they are still tied to the magnetic field lines. For the plasma to detach from the MN, both the ions and electrons have to be deviated from the field lines to maintain zero net current exhausted from the system. Otherwise, self-induced electric fields pulling back the ions will develop. One experiment on the plasma in the MN has discussed the anomalous transport of the electrons due to wave generation, where the wave was considered to induce an “outward” radial electron transport, resulting in the plasma loss from the MN and degrading the thruster performance^24^. Plasma detachment is also an important problem relevant to solar physics, i.e., the characterization of the boundary between the Sun’s magnetized atmosphere and interstellar space, where turbulent signals have recently been measured^25^. The detached plasmas escape from being governed by the solar magnetic fields to form the solar wind ejected toward surrounding planets including the Earth. In the magnetosphere, a whistler wave and a magnetosonic wave (equivalent to a compressional Alfvén wave) can heat the electrons to high energies^26–28^ and also affect the plasma transport via pitch angle scattering and cross-field transport of charged particles^29,30^. Plasma transport and loss due to waves, instabilities, and turbulence have been one of the greatest problems for plasma confinement in thermonuclear fusion reactors^31^.

For the present experiment, the cross-field “inward” electron transport induced by the spontaneously generated wave in the MN is demonstrated by considering a nonlinear interaction between the density and velocity fluctuations. The measured dispersion relation well fits that of the magnetosonic wave. Since the major component of the electric field for the wave mode is parallel to the wavenumber in the detected frequency range, being much higher than the ion cyclotron frequency and close to the lower hybrid frequency, it behaves as an electrostatic mode and the electric field fluctuation drives the cross-field transport only for the electrons. Simultaneously, the spatial ion velocity mapping shows the ions directed preferentially toward the main axis, compared to the divergent magnetic field lines, which is driven by the electrostatic acceleration due to the steady-state electric field. Therefore, it can be deduced that the wave-driven, cross-field, inward electron transport plays a significant role in maintaining the current balance between the electrons and the ions detached from the magnetic field lines.

## Results and discussion

**Figure 1:**
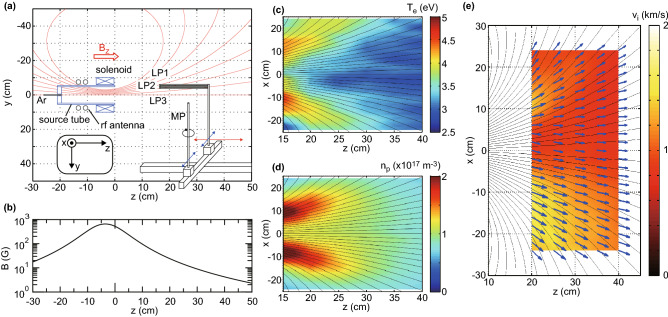
Experimental setup and the measured electron temperature, plasma density, and ion velocity in steady state. (**a**) Schematic diagram of the experimental setup. (**b**) The calculated magnetic field strength on axis, showing the divergent magnetic field. Measured *x*–*z* profiles of (**c**) the electron temperature $T_e$, (**d**) the plasma density $n_p$, and (**e**) the vector (arrows) and magnitude (colored scaling) of the ion velocity ${\mathbf{v}}_i$, together with the calculated magnetic field lines (solid lines). The conical structures of $T_e$ and $n_p$ are formed near the thruster exit and the $n_p$ profile becomes nearly parabolic downstream, where the large amplitude wave is detected. The divergence of the ions are found to be smaller than that of the magnetic field lines, indicating the deviation of the ions from the magnetic field lines.

**Figure 2:**
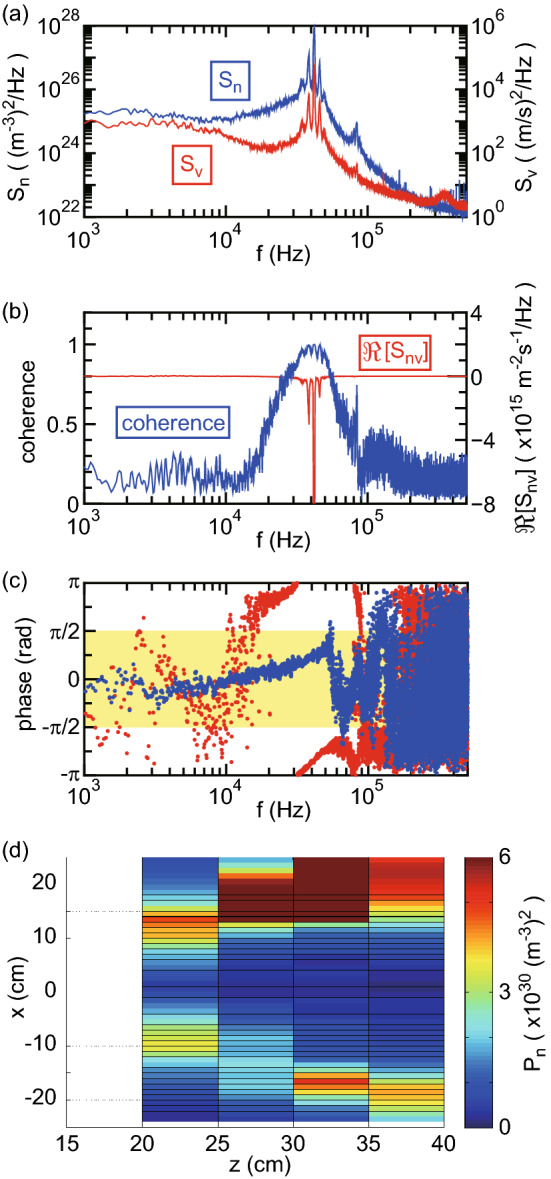
Frequency analyses of the density and velocity fluctuations. (**a**) Typical power spectrum densities (PSDs) of $\tilde{n}_p(t)$ ($S_n$: blue line) and $\tilde{v}_e(t)$ ($S_v$: red line) taken at $(x,z)=(18~{\rm{cm}}, 30~{\rm{cm}})$. The large amplitude fluctuations in $\tilde{n}_p(t)$ and $\tilde{v}_e(t)$ can be found at around 40 kHz. (**b**) The coherence and the real part of the cross spectrum $\Re [S_{nv}]$ of the $\tilde{n}_p(t)$ and $\tilde{v}_e(t)$ signals in (**a**). Strong correlation between $\tilde{n}_p(t)$ and $\tilde{v}_e(t)$ can be confirmed by the coherence and the cross-field inward electron flux induced by the fluctuation and directed to the negative *x* direction can be identified by $\Re [S_{nv}]$. (**c**) The cross-phase between $\tilde{n}_p(t)$ and $\tilde{v}_e(t)$ taken at $(x,z)=(18~{\rm{cm}}, 30~{\rm{cm}})$ (red dots) and $(x,z)=(-18~{\rm{cm}}, 30~{\rm{cm}})$ (blue dots), where the yellow-colored region shows that the flux is directed to the positive *x* direction. The phase around 40 kHz clearly demonstrates that the electron flux is directed to the negative and positive *x* directions at $x=18~{\rm{cm}}$ and $x=-18~{\rm{cm}}$, respectively. (**d**) *x*–*z* profile of the power $P_n$ of the density fluctuation obtained by integrating $S_n$ over the frequency. The fluctuation is found to exist at the peripheral region of the plasma flow along the MN.

**Figure 3:**
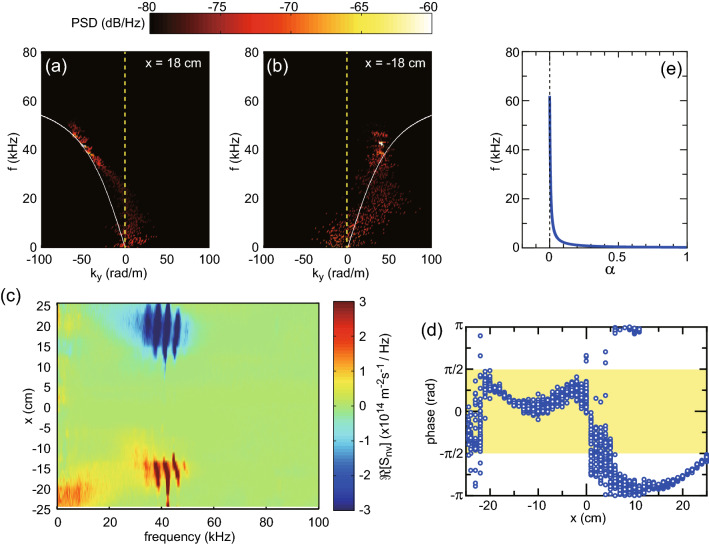
Dispersion curves of the wave and the cross-field electron flux. (**a**) The PSD mappings (colored scale) of the $V_{f1}$ signal on the $k_y$-*f* diagrams for (**a**) $(x,z)=(18~{\rm{cm}}, 30~{\rm{cm}})$ and (**b**) $(-18~{\rm{cm}}, 30~{\rm{cm}})$, together with the dispersion curve of the magnetosonic wave propagating perpendicular to the magnetic field, where the dashed lines show $k_y=0$. The fluctuation propagates in the azimuthal direction and is identified as the magnetosonic wave. (**c**) *x* profile of $\Re [S_{nv}(f)]$, being equivalent to the wave-driven and frequency-decomposed cross-field electron flux. The cross-field inward electron flux is driven by the magnetosonic wave around 40 kHz. (**d**) *x* profile of the cross-phase between $\tilde{n}_p(t)$ and $\tilde{v}_e(t)$ for the frequency range of $42\pm 1$ kHz, together with the yellow-colored region corresponding to the flux directed to the positive *x* direction. The phase data identifies the inward electron flux toward the main axis as well as $\Re [S_{nv}(f)]$ in (**c**). (**e**) The calculated ratio of $\alpha = |E_{\perp }/E_{\parallel }|$ for the dispersion branch in (**a**, **b**), where $E_{\parallel }$ and $E_{\perp }$ are the electric fields parallel and perpendicular to the wavenumber, respectively. The calculated $\alpha$ close to zero in the frequency range higher than $f_{ci}$ and lower than $f_{LH}$ implies that the magnetosonic wave branch behaves like an electrostatic mode.

The experiment is performed using a magnetized radiofrequency (rf) plasma source immersed in a 1-m-diameter and 2-m-long vacuum chamber evacuated by a combination of three turbomolecular pumps to a base pressure of less than $10^{-4}$ Pa (Fig. 1a). The source consists of a 9.5-cm-inner-diameter, 11-cm-outer-diameter, and 20-cm-long ceramic source tube surrounded by a double-turn rf loop antenna, and a solenoid providing the MN having a maximum strength of about 700 G at the solenoid center. The calculated magnetic field lines and strength along the *z* axis are shown in Fig. 1a, b, respectively, where the origin of the Cartesian coordinate system shown by the inset in Fig. 1a is the radial center of the source open exit. The upstream side of the source tube is terminated by an insulator plate ($z=-18.5$ cm) having a small center hole, where argon gas is introduced. The gas flow rate is maintained at 70 sccm, giving a chamber pressure of 28 mPa; the mean free path for the electron-neutral elastic collisions is about 1.5 m and larger than the axial and radial plasma scales. The rf antenna is powered by a 13.56 MHz rf generator via an impedance matching circuit located outside the chamber and a vacuum feedthrough on the chamber side wall at the negative *x* position. The rf power is presently set at 1.5 kW and turned on for 0.5 s, with the capacitors in the matching circuit tuned in advance to minimize rf power reflection (indeed no measurable power reflection was detected).

Three cylindrical Langmuir probes (LPs) aligned in the *y* direction (top, middle, and bottom LPs labelled LP1, LP2, and LP3 on Fig. 1a, respectively) at intervals of 4 mm are mounted on a movable motor stage and the ion saturation current ($I_{is}$) at constant probe bias voltage of $-90$ V and the electron temperature ($T_e$) are estimated from the LP2 current/voltage characteristic. The plasma density ($n_p$) is derived from the measured $I_{is}$ and $T_e$ as $I_{is}=0.61 e n_p u_B S$, where $u_B$ is the Bohm velocity which depends on $T_e$ and the ions are assumed unmagnetized. The *x*–*z* profiles of $T_e$ and $n_p$ are presented in Fig. 1c,d, respectively, and exhibit the characteristic spatial mapping of the high temperature electrons and the high density conics in the peripheral region near the thruster exit as observed previously^32–35^; the density profile becomes nearly parabolic further downstream at $z\sim 30$ cm. The slight asymmetries with respect to the *z* axis can be seen in the $n_p$ and $T_e$ mappings and seem to originate from the high-voltage antenna terminal existing near the source wall, where the high rf voltage is coupled near the wall as observed previously^36^. Therefore, all the measurements are performed over both the negative and positive *x* regions in the present experiment.

The Mach probe (MP) mounted on a rotational stage (see Fig. 1a) is used to measure the axial (*z*) and horizontal (*x*) ion Mach numbers^37^, which corresponds to the averaged ion velocity. By taking the $T_e$ profile in Fig. 1c into account, the magnitude and direction of the local ion velocity ${\mathbf{v}}_i$ can be obtained from the measured Mach numbers and are shown by colored scaling and arrows in Fig. 1e, respectively. The maximum velocity is about 1.6 km/s corresponding to a Mach number of about 0.5. A number of experiments have shown the generation of a supersonic ion beam in the measured ion energy distributions. This ion beam component is generated by the spontaneously formed steady-state electric field near the source exit, which has axial and radial components. A part of the beam component is converted into fast neutrals via charge exchange collisions and thermal ions newly appears in the MN^9,38^. The mean free path for the ion-neutral charge exchange collisions is about 50 cm and effectively acts as a drag force for the accelerated ions, resulting in the Mach number lower than unity in the averaged velocity measurement by the MP. However, the considerable deviation of the averaged ion velocity from the magnetic field lines can be confirmed here since the divergence angles of the ion velocity vectors are smaller than those of the magnetic field lines. Since the typical ion Larmor radius in the MN is larger than the radial scale of the plasma, the ion orbits are affected by the steady-state electric field rather than the magnetic field. The cross-field ion flux deviation from the MN in the *r*–*z* (or *x*–*z*) plane appears clearly on Fig. 1e over the whole region of the nozzle investigated.

Insight into the transport of electrons in the diverging nozzle region can be obtained by determining the cross-field continuous electron flux $\Gamma _{e\perp }$. In plasmas, this flux can be produced by a nonlinear term combining the density $\tilde{n}_p(t)$ and velocity $\tilde{v}_e(t)$ fluctuations, where the electron cross-field velocity is given by $\tilde{v}_e = \tilde{E}_{\theta }/B$ ($\tilde{E}_{\theta }$ is the azimuthal electric field fluctuation, which corresponds to $\tilde{E}_y/B$ on the *z*–*x* plane). It can be described using either the temporally varying or the frequency(*f*)-decomposed forms^39^:

1 $$\begin{aligned} \Gamma _{e\perp }= & {} \frac{1}{T}\int _0^T \tilde{n}_p(t)\tilde{v}_e(t) dt = \int \Re \left[ {S_{nv}(f)} \right] df, \end{aligned}$$

where $S_{nv}(f)$ is the one-sided cross spectrum of $\tilde{n}_p(t)$ and $\tilde{v}_e(t)$ and $\Re \left[ {S_{nv}(f)} \right] >0$ indicates the cross-field electron flux pointing in the positive *x* direction. The direction of the cross-field electron flux can also be identified by the phase of $S_{nv}$ as well as the sign of $\Re \left[ {S_{nv}(f)} \right]$. By considering the direction of the **E** × **B** drift, the phase between $\pm \pi /2$ shows the flux pointing in the positive *x* direction. $I_{is}$ from LP2 and the floating potentials $V_{f1}$ and $V_{f3}$, respectively from LP1 and LP3, are simultaneously measured at a sampling rate of 1 MS/s and 12 bit resolution. $\tilde{n}_p(t)$ and $\tilde{v}_e(t)$ are derived from $I_{is}$ and $(V_{f1}-V_{f3})/(Bd)$, where $d=8$ mm is the distance between LP1 and LP3. Here the temperature fluctuation is assumed to be negligible for the density and potential measurements and for the transport calculation, as previously validated in low-temperature plasmas^40^.

Typical power spectrum densities (PSDs) of the density ($S_n$) and the electron **E** × **B** drift velocity ($S_v$) taken at $(x,z)=(18~{\rm{cm}}, 30~{\rm{cm}})$, and the coherence of these signals are shown in Fig. 2a,b, respectively. Both PSDs exhibit a very strong frequency component at around 40 kHz as seen in Fig. 2a and the coherence at that frequency is close to unity as seen in Fig. 2b. Therefore, the strong correlation between the density and velocity fluctuations at around 40 kHz is confirmed. The real part of the cross-spectrum $\Re [S_{nv}(f)]$ equivalent to the frequency-decomposed cross-field electron flux is shown on the right-hand axis by a red solid line in Fig. 2b and shows the negative value at around 40 kHz. The negative value of $\Re [S_{nv}(f)]$ at the positive *x* position indicates the electron flux pointing in the negative *x* direction and the detailed profile will be described later. The cross phase between $\tilde{n}_p(t)$ and $\tilde{v}_e(t)$ taken at $(x,z)=(18~{\rm{cm}}, 30~{\rm{cm}})$ and $(x,z)=(-18~{\rm{cm}}, 30~{\rm{cm}})$ are plotted in Fig. 2c by red and blue dots, respectively. At around 40 kHz, the phase is out of and within the range of $\pm \pi /2$ at $x=18$ cm and $x=-18$ cm, respectively, indicating the cross-field fluxes pointing in the center axis. Figure 2d shows the *x*-*z* profile of the power $P_n$ of the density fluctuation obtained by integrating the PSD $S_n$. The $P_n$ power mapping over the experimentally investigated axial region shows that the fluctuation exists at the peripheral region along the MN with a largest amplitude observed near $z\sim 30$ cm, where the density profile is close to parabolic or uniform rather than conical as seen in Fig. 1d.

The cross spectrum of the measured $V_{f1}$ and $V_{f3}$ yields their phase difference as a function of frequency (*f*) and corresponds to the wavenumber $k_y$ in the *y* direction; the PSD is simultaneously calculated from the $V_{f1}$ data to access information on the wave amplitude. PSDs magnitudes as a function of ($k_y$, *f*) obtained from the data taken at (*x*, *z*) = (18 cm, 30 cm) and (− 18 cm, 30 cm) are shown by colored scaling in Fig. 3a,b, respectively. The results show maximum amplitude at around $(k_y, f) \sim (\pm 50~\mathrm{rad/m},~40~\mathrm{kHz})$. It is clearly seen that the $k_y$ measured at $x=\pm 18$ cm have similar absolute values and opposite signs, demonstrating that the wave propagates in the azimuthal direction. The estimated $k_y$ implies a high-order azimuthal mode number close to 10 and the sidebands around 40 kHz seem to represent other azimuthal mode number components. To further ascertain the measured results shown in Fig. 3a,b, a comparison with the dispersion relation of a wave propagating perpendicularly to the magnetic field in a uniform and cold plasma is carried out by considering the (*S*, *D*, *P*) elements of the dielectric tensor following Stix’s notation^41^:

2 $$\begin{aligned}{}&(S\sin ^2\theta +P\cos ^2\theta )N^4 - \nonumber \\&\quad \left[ RL \sin ^2\theta +PS(1+\cos ^2 \theta ) \right] N^2 + PRL = 0, \end{aligned}$$

where $R=S+D$, $L=S-D$, $\theta$ is the wavenumber angle to the magnetic field, and $N=c k /\omega$ is the refractive index. The direct comparison between the measurements at (*x*, *z*) = (±18 cm, 30 cm) and the theory is performed using the measured local plasma density of $n_p=9\times 10^{16}$ m$^{-3}$ and the magnetic field strength of 6 G. For these parameters and the typical electron temperature of $T_e\sim 3.5$ eV, the ion cyclotron frequency, the lower hybrid frequency, the Alfvén speed, and the ion sound speed, are $f_{ci}\sim 0.25$ kHz, $f_{LH}\sim 60$ kHz, $v_A\sim 6.9$ km/s, and $C_s\sim 3$ km/s, respectively. It should be noted that a finite electron temperature mainly affects a damping or growth rate of waves, i.e., an imaginary part of the frequency, and has little impact on the $\omega$-*k* diagram dispersion relation^42,43^. In fact, the dispersion relation assuming cold plasmas has been well described and fitted to observations of the low-frequency waves, e.g., in Ref.^44^. The white solid lines in Fig. 3a,b represent the calculated dispersion relation for $\theta =\pi / 2$ in radian, i.e., the dispersion branch corresponding to the magnetosonic wave, and are a good fit of the ($k_y$, *f*) measurements. Therefore, the fluctuation at around 40 kHz can be considered to be the magnetosonic wave propagating in the azimuthal direction, where the wave spectra become discrete so as to satisfy both the dispersion relation and the azimuthal continuity of the electric fields.

Figure 3c shows the *x* profile of $\Re [S_{nv}(f)]$ taken at $z=30$ cm, i.e., the frequency-decomposed electron flux perpendicular to the magnetic fields, where the measurements are taken horizontally at 1 cm intervals. The negative and positive values of $\Re [S_{nv}(f)]$ around 40 kHz appear at around $|x|=$15–20 cm in the positive and negative *x* regions, respectively. The phase data at the frequency range of $41\pm 1$ kHz, where the large wave amplitude is detected, are extracted from $S_{nv}$ and plotted in Fig. 3d, where the yellow-colored region shows the flux pointing in the positive *x* direction. Since both $\Re [S_{nv}]>0$ and the phase within $\pm \pi /2$ (the yellow region) indicate the cross-field flux in the positive *x* direction as already described, the result in Fig. 3c,d implies that the electron flux at $|x|=$15–20 cm toward the center axis is produced by the magnetosonic wave around 40 kHz.

The cross-field electron flux in Fig. 3c is driven by the azimuthal electric field (corresponding to $\tilde{E}_y$) of the wave propagating in the azimuthal direction, implying that the wave is likely an electrostatic mode. The ratio of $\alpha = |E_{\perp }/E_{\parallel }|$ for the dispersion branch of the magnetosonic wave can be derived from a wave equation as

3 $$\begin{aligned} \alpha= & {} \left| \frac{E_{\perp }}{E_{\parallel }} \right| = \left| \frac{iD}{S-N^2} \right| , \end{aligned}$$

where $E_{\parallel }$ and $E_{\perp }$ are the electric fields parallel and perpendicular to the wavenumber, respectively. The calculated $\alpha$ is drawn in Fig. 3e and is found to be close to zero at the frequency range of $f_{ci} \ll f < f_{LH}$, i.e., the electric field parallel to the wavenumber is much larger than that perpendicular to the wavenumber. Therefore, the magnetosonic wave in the detected frequency range behaves like an electrostatic wave mode and does not have significant magnetic field fluctuation, being consistent with the experimental observation of the presence of the azimuthal electric field fluctuation and wavenumber. Based on this consideration, it can be concluded that the cross-field electron transport is mainly induced by the electric field of the magnetosonic wave.

**Figure 4:**
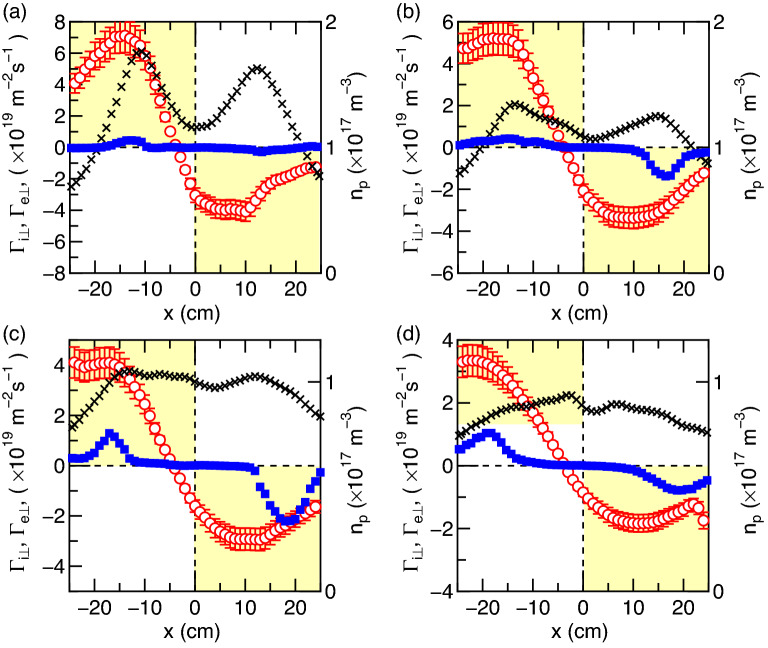
Cross-field fluxes of the electrons and ions. *x* profiles of the fluxes perpendicular to the magnetic field lines for the ions ($\Gamma _{i\perp }$, open circles) and the electrons ($\Gamma _{e\perp }$, filled squares) at (**a**) $z=20$ cm, (**b**) $z=25$ cm, (**c**) $z=30$ cm, and (**d**) $z=35$ cm, together with the plasma density ($n_p$, crosses). The errors in $\Gamma _{e\perp }$ and $n_p$ estimated by repeating 100 shots are about $2~\%$ for both those respective data sets and smaller than the marker sizes. Detailed error analysis is shown in the ’Method’ section. The yellow-colored regions indicate the radially inward cross-field transport. The cross-field transport by the magnetosonic wave for the electrons drives the inward electron flux overcoming the pressure gradient force and contributes to the current balance with the ions deviating from the magnetic field lines.

The ion flux $\Gamma _{i\perp }$ perpendicular to the magnetic field lines can be estimated from $n_p$ in Fig. 1d, ${\mathbf{v}}_i$ in Fig. 1e, and the magnetic fields ${\mathbf{B}}$ as $\Gamma _{i \perp } = n_p |v_i| \sin (\theta _v-\theta _B)$, where $\theta _v$ and $\theta _B$ are the angles of ${\mathbf{v}}_i$ and ${\mathbf{B}}$ to the *z* axis, respectively. Conversely, the wave-induced electron flux $\Gamma _{e\perp }$ perpendicular to the magnetic field can be calculated according to Eq. (1) and the horizontal profile of $\Re [S_{nv}(f)]$. The *x* profiles of the fluxes $\Gamma _{i\perp }$ and $\Gamma _{e\perp }$ taken at (a) $z=20$ cm, (b) 25 cm, (c) 30 cm, and (d) 35 cm, are plotted by open circles and filled squares in Fig. 4, respectively. The yellow and white regions in the panes of Fig. 4 correspond to the fluxes inward and outward the *z* axis, respectively. Both the ion and electron fluxes show less divergent transport than the magnetic field lines, the former induced by the steady-state electric fields near the source exit and the latter by the cross-field inward transport due to the magnetosonic wave. It can be seen that the electron flux is close to a few tens of percent of the ion flux in the peripheral region, indicating that the wave-driven inward transport process for electrons makes a contribution to the current balance with the ions detached from the magnetic fields.

As observed previously, the unmagnetized ions accelerated by the steady-state electric fields having both the axial and radial components do not perfectly follow the magnetic field lines; the electrostatic ion acceleration induces both the ion fluxes parallel and perpendicular to the magnetic field lines in the *r*–*z* plane^9^. The ions deviating from the field lines seen in Fig. 1e can carry some sort of current across the magnetic field, while the cross-field electron current can be induced by the wave-driven transport as shown by the present experiment. Since the directions of the ion deviation from the field lines and of the wave-driven electron transport are the same, the cross-field electric current can be mitigated. As the total net current should be zero in the current-free rf plasma source, the electric current parallel to the field lines should also be zero. The previous measurement of the electron energy probability functions has clearly demonstrated that the energetic electrons overcoming the parallel electric fields can neutralize the accelerated ions^45^, whereby zero net current is maintained along the field lines.

To discuss the energy source driving the wave, the radial density profiles are plotted by crosses in Fig. 4. It is found that the maximum wave-driven flux appears at the peripheral region where the radial density gradient is negative as clearly seen in Fig. 4c,d. In general, the flux induced by the pressure-gradient-driven mode attempts to mitigate the energy source, i.e., the pressure gradient, while the wave-driven transport induces an electron flux overcoming the pressure gradient force in the present experiment. The measured radial potential profile at $z=30$ cm is nearly uniform or parabolic (not shown here) and an **E** × **B** induced instability such as a Simon–Hoh type is stable for this case^46^. Therefore, the energy source of the wave is neither the pressure gradient nor the potential gradient. Previous studies have shown that the magnetosonic wave in a frequency range of $f_{ci}< f < f_{LH}$ can be excited by the presence of ions accelerated perpendicular to the magnetic fields^26,47^, where the likely energy source of the wave is the ion energy across the magnetic field lines. Our measurement on the ion velocity mapping (Fig. 1e) clearly shows the presence of the ions deviating from the magnetic field lines in the *r*–*z* plane, where the cross-field ion flux is maximized at the peripheral region. Therefore, it can be deduced that the energy of the ions deviating from the magnetic field lines is the energy source of the magnetosonic wave. The wave-induced inward electron flux attempts to neutralize and mitigate the ions deviated from the field lines, where the deviation of the unmagnetized ions is due to the presence of the steady-state axial and radial electric fields as observed in a number of experiments, e.g., Ref.^9^. Based on these considerations, it is considered that the magnetosonic wave and electron inward transport could occur in various types of plasma flows in a MN where the unmagnetized ions deviate from the field lines.

The ions in the MN are indeed unmagnetized here; the driving force for the ion acceleration and the ion flux deviating from the magnetic field lines is the steady-state electric fields. When a part of the ion energy is consumed to generate the wave, the ion energy would be damped. As the fluctuation much higher than $f_{ci}$ does not directly affect the cross-field ion transport, other physical process for damping the ion energy will be required. One possible scenario is the reduction of the steady-state electric field accelerating the ions. Verification of such a drag force for the ions would require an extremely accurate ion velocity and plasma potential measurements and an accurate theoretical model. These remain further challenging issues.

**Figure 5:**
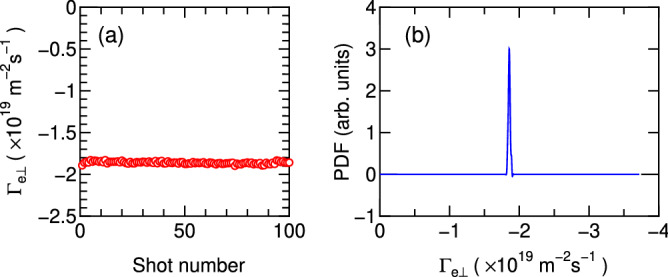
Error analysis of the cross-field electron flux. (**a**) The cross-field electron flux $\Gamma _{e\perp }$ as a function of the shot number and (**b**) the probability density function (PDF) obtained from Fig. 5a. These shows the significantly small error of about $\pm 2~\%$ in the measurement.

## Conclusion

In the plasma expanding in the MN, where the ions are accelerated by the steady-state electric field, are unmagnetized, and deviate from the field lines, fluctuations having a frequency less than the lower hybrid frequency and much larger than the ion cyclotron frequency are detected and identified as the magnetosonic wave propagating primarily in the azimuthal direction. In the detected frequency range, the dispersion branch of the magnetosonic wave is likely an electrostatic wave having the electric field fluctuation parallel to the wavenumber. It is demonstrated that the nonlinear interaction between the density fluctuation and the electric-field-driven velocity fluctuation related to the magnetosonic wave induces an inward cross-field electron transport toward the main axis of the MN. The electron cross-field transport plays a significant role in the current balance with the accelerated ions detached from the magnetic field lines, which could be a common phenomenon in terrestrial plasma devices and naturally occurring plasmas.

## Methods

**Error analysis of the cross-field electron transport**

To assess the possible error in the wave-driven cross-field electron flux, the signals of the ion saturation current $I_{is}(t)$ from LP2 and the floating potentials $V_{f1}$ and $V_{f3}$ from the LP1 and LP3 are taken by repeating 100 shots at $z = 30$ cm and $x=20$ cm. Figure 5a shows the cross-field electron flux $\Gamma _{e\perp }$ as a function of the shot number, clearly showing good reproducibility. The probability density function (PDF) shown in Fig. 5b is obtained from the data in Fig. 5a. The PDF has very narrow peak around $\Gamma _{e\perp }= -1.86 \times 10^{19}$ m$^{-2}$s$^{-1}$ with a standard deviation of $\sigma = 1.38\times 10^{17}$ m$^{-2}$s$^{-1}$. Even if we consider the error as $\pm 3\sigma$, the error is only about $\pm 2~\%$ of the averaged value and smaller than the marker size in Fig. 4.

## Data Availability

The datasets used and/or analyzed during the current study are available from the corresponding author on reasonable request.
